# Biodiversity–stability relationships strengthen over time in a long-term grassland experiment

**DOI:** 10.1038/s41467-022-35189-2

**Published:** 2022-12-14

**Authors:** Cameron Wagg, Christiane Roscher, Alexandra Weigelt, Anja Vogel, Anne Ebeling, Enrica de Luca, Anna Roeder, Clemens Kleinspehn, Vicky M. Temperton, Sebastian T. Meyer, Michael Scherer-Lorenzen, Nina Buchmann, Markus Fischer, Wolfgang W. Weisser, Nico Eisenhauer, Bernhard Schmid

**Affiliations:** 1 Department of Geography, Remote Sensing Laboratories, University of Zürich, Winterthurerstrasse 190, CH-8057 Zürich, Switzerland; 2 Fredericton Research and Development Centre, Agriculture and Agri-Food Canada, 95 Innovation Road, Post Office Box 20280, Fredericton, E3B 4Z7 NB Canada; 3 UFZ, Helmholtz Centre for Environmental Research, Department of Physiological Diversity, Permoserstrasse 15, D-04318 Leipzig, Germany; 4 German Centre for Integrative Biodiversity Research (iDiv) Halle-Jena-Leipzig, Puschstraße 4, 04103 Leipzig, Germany; 5 Institute of Biology, Leipzig University, Johannisallee 21, 04103 Leipzig, Germany; 6 Institute of Ecology and Evolution, University of Jena, Dornburger Straße 159, D-07743 Jena, Germany; 7 Institute of Plant Sciences, University of Bern, Altenbergrain 21, 3013 Bern, Switzerland; 8 Institute of Ecology, Leuphana University, Lüneburg, Germany; 9 Terrestrial Ecology Research Group, Department of Ecology and Ecosystem Management, Center for Food and Life Sciences Weihenstephan, Technische Universitat Munchen, Hans-Carl-von-Carlowitz-Platz 2, D-85350 Freising-Weihenstephan, Germany; 10 Geobotany, Faculty of Biology, University Freiburg, Schänzlestr. 1, D-79104 Freiburg, Germany; 11 Institute of Agricultural Sciences, ETH Zurich, Universitatstrasse 2, 8092 Zurich, Switzerland; 12 Institute of Biology, Leipzig University, Puschstraße 4, 04103 Leipzig, Germany

**Keywords:** Community ecology, Grassland ecology, Biodiversity

## Abstract

Numerous studies have demonstrated that biodiversity drives ecosystem functioning, yet how biodiversity loss alters ecosystems functioning and stability in the long-term lacks experimental evidence. We report temporal effects of species richness on community productivity, stability, species asynchrony, and complementarity, and how the relationships among them change over 17 years in a grassland biodiversity experiment. Productivity declined more rapidly in less diverse communities resulting in temporally strengthening positive effects of richness on productivity, complementarity, and stability. In later years asynchrony played a more important role in increasing community stability as the negative effect of richness on population stability diminished. Only during later years did species complementarity relate to species asynchrony. These results show that species complementarity and asynchrony can take more than a decade to develop strong stabilizing effects on ecosystem functioning in diverse plant communities. Thus, the mechanisms stabilizing ecosystem functioning change with community age.

## Abstract (web summary)

Biodiversity-ecosystem functioning relationships may change over time. Here, Wagg et al. show that richness-productivity and richness stability relationships grow stronger over time in an experimental grassland community, and shed light on the ecological mechanisms.

## Introduction

Decades of empirical and theoretical research have shown that a greater number of species enhances the productivity of an ecosystem, in the short-term, and can sustain higher levels of productivity in the long-term^1–7^. However, the effects of diversity on ecosystem functioning can change over years, whereby the positive effect of species richness on ecosystem functioning often becomes stronger with time in experimental communities following initial establishment^8–12^. Consequently, there has been a growing interest as to why biodiversity–ecosystem functioning relationships change through time and the underlying mechanisms by which species richness maintains a more stable ecosystem functioning^13–15^. There are few long-term studies able to experimentally address such long-term temporal patterns. The experiments that exist have demonstrated that species richness–ecosystem functioning relationships can strengthen over the years because of the various demographic and evolutionary processes that take place; such as species turnover and local selection to avoid competition that can thus lead to complementary resource use^4,8,11,12,14,16–18^. Regardless of the underlying processes, these studies all illustrate the temporal importance of biodiversity for sustaining ecosystem functioning. This can be attributed to an increasing complementarity effect (CE)^19–21^ among species through time, whereby species are, on average able to maintain, or even increase, their productivity over many years in mixtures better than in monoculture (e.g., by resource partitioning, facilitation, or biotic interactions^22^). By maintaining greater temporal productivity, more diverse communities also maintain more stable productivity. Thus, the temporally increasing biodiversity–productivity relationships should lead to increasing biodiversity–stability relationships and its underlying mechanisms over time, which has not yet been tested in biodiversity experiments.

The ability of a community to maintain temporally stable productivity across multiple years is captured by the inverse of the coefficient of variation (CV^−1^: the temporal standard deviation relative to the mean) of community productivity^3^. Past long-term grassland biodiversity experiments have shown that greater species richness can maintain more stable productivity due to greater insurance that some species will be able to maintain productivity during times when others cannot, such as during a drought or other disturbances, referred to as portfolio or insurance effect^1,23^. Thus, plant community productivity is stabilized by species that are temporally asynchronous in their performance as well as by the presence of particularly productive species that exhibit stable population dynamics through time^24–26^. Furthermore, high community productivity and overyielding in species mixtures (i.e., mixtures yielding more than the average of their species grown in monocultures) can stabilize community productivity^26–29^.

While it has been documented that species richness–productivity relationships strengthen over time in biodiversity experiments^8,10,11^, it has not been assessed whether species richness–stability (of productivity) relationships do also; and if they do, what the contribution of the three mechanisms mentioned above—asynchrony, population stability, and overyielding—would be. Furthermore, linkages between these mechanisms stabilizing community productivity and the temporal dynamics of biodiversity effects, in particular the mentioned complementarity effect (CE), have been little explored^20,21^. This is largely because there are few long-term studies that can address such questions.

Here, we assessed the change in species richness–productivity and richness–stability relationships over 17 years in a long-term grassland biodiversity experiment, the Jena Experiment^13^. We hypothesize that the species richness–productivity relationship strengthens over time due to increasing CEs, but also that these increasing biodiversity effects and CEs at the same time can strengthen the species richness–stability relationship over time. For instance, the species richness–productivity relationship may strengthen due to declining monoculture productivity and thus increasing CEs. The resulting maintenance of relatively greater productivity at higher diversity levels may also temporally increase the positive effect of diversity on stability. In addition, a strengthening of the CE through time could also indicate an increase in the temporal niche segregation among species to avoid competition, and thus lead to more stable population dynamics of the species, again contributing to increased stability. Finally, increasing species asynchrony over time could also reflect yearly varying selection effects (SEs, where more diverse communities have a greater probability to contain species that dominate and have a strong effect on ecosystem functions^19^). These annual SEs could scale up to an interannual CE when different species dominate the community among years^30^, thus, over time, increasingly stabilizing productivity in diverse communities through increasing asynchrony^20,21^. Here we test these hypotheses about temporally changing species richness–productivity, –complementarity, –stability, and –asynchrony relationships using data from the Jena Experiment^13^. Results of our study show that species richness increasingly supported higher productivity over 17 years, due to increasing CEs among species in more species-rich communities. Consequently, greater species richness-driven CEs had an increasingly positive effect on stabilizing the community productivity over time. Further, we found that only after the first decade of the experiment did the CE also stabilize the community productivity through a positive effect on species asynchrony. Together these results show that the underlying mechanisms of community stability, namely species asynchrony and population stability, and overyielding-related CEs, are also temporally dynamic.

## Results

### Temporal change in biodiversity–productivity relationships

**Fig. 1:**
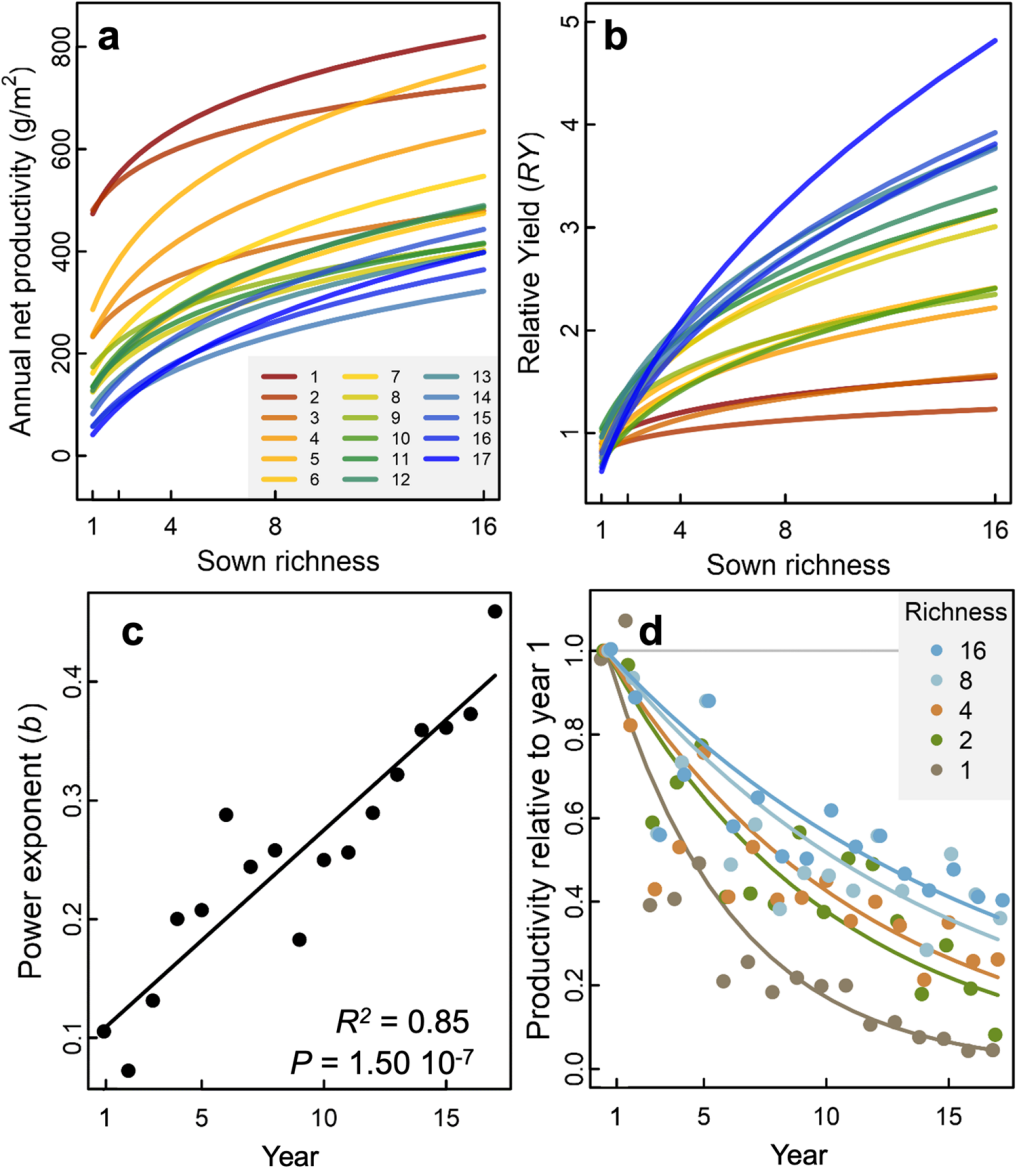
Sown species richness–productivity relationships through time. The log-linear relationships between sown species richness and **a** aboveground net primary productivity (ANPP square root transformed prior to analysis) of the communities and **b** relative yield (ANPP divided by mean ANPP of monocultures in that year) of the communities are shown for each year (1 = 2003, 17 = 2019). **c** The slope of the log–log relationship (power exponent *b* of curves shown in **b**) corresponding to the increase in biomass per added species relative to the mean ANPP of all monocultures for each year. **d** The change in ANPP of the communities over time relative to their ANPPs in year 1 for each sown species richness level 1–16.

Over the years, the aboveground net primary productivity (ANPP) of all communities generally declined (Fig. 1a, d and Fig. S1). Greater species richness consistently resulted in greater ANPP. The positive effect of richness on ANPP increased significantly over the 17-year period (log-richness by linear-year interaction: *F*_1, 329.9_ = 8.34, *P* = 0.004, Table S3), reflected in an increasingly steeper richness–productivity slope (see Table S4). Similarly, the slope of the species richness–relative yield (RY*,* ANPP divided by mean ANPP of monocultures in that year) relationship became increasingly steeper and less saturating over the years (log-richness by linear-year interaction: *F*_1, 331.1_ = 44.29, *P* < 0.001, Fig. 1b, c and Table S3). Productivity declines relative to the first year were steepest for monocultures and low-diversity mixtures and flatter for high-diversity mixtures (year by richness-as factor: *F*_4, 244.3_ = 13.79, *P* < 0.001, Table S5). This revealed that the strengthening effect of species richness on productivity increased over the 17-year period because of a greater decline in monocultures relative to more diverse plant communities, with the 16-species mixtures still declining, but declining the least (Fig. 1d).

### Temporal change in biodiversity effects

**Fig. 2:**
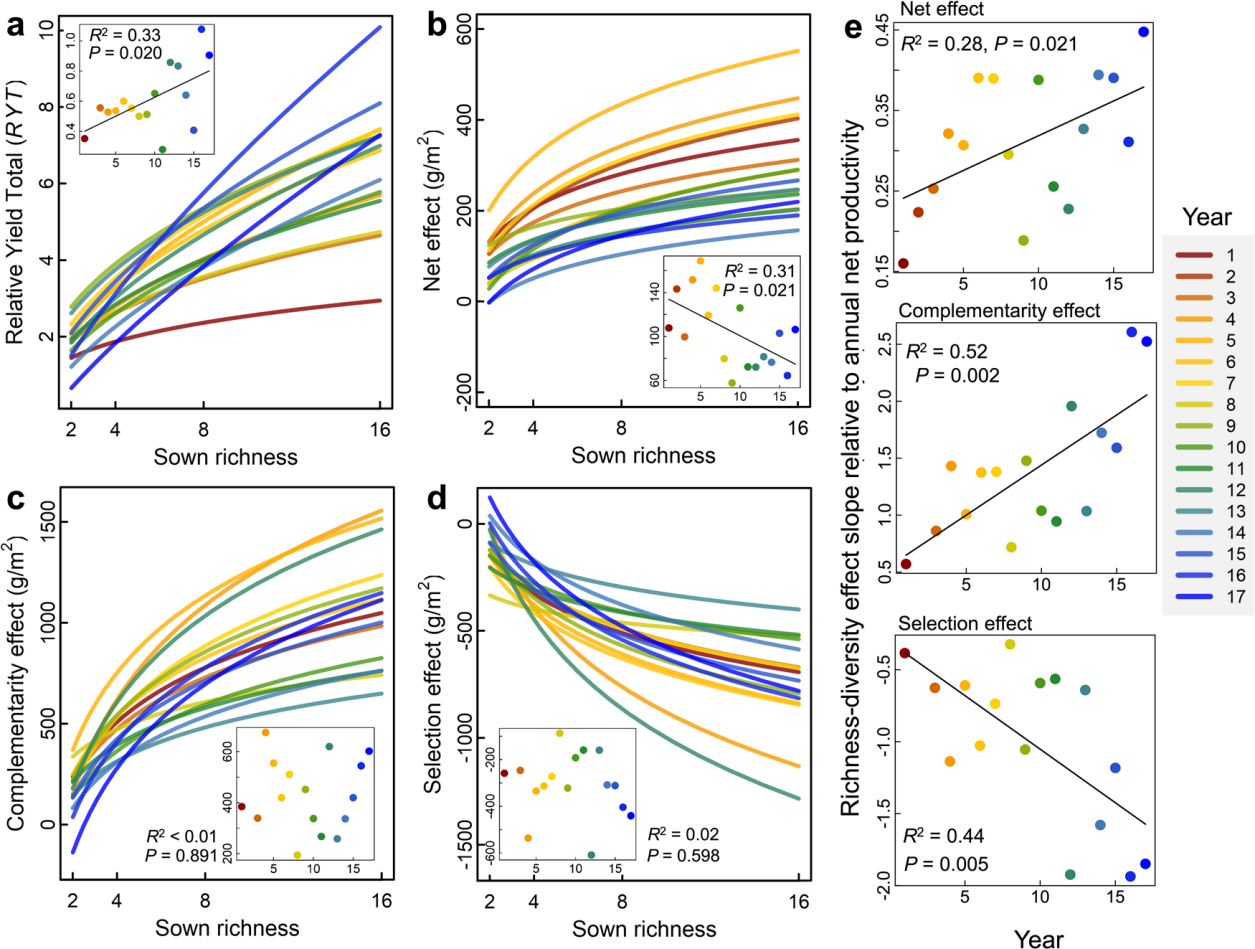
Effects of sown species richness on the annual biodiversity effects. The effects of richness on the **a** relative yield total (RYT), **b** net, **c** complementarity, and **d** selection biodiversity effects are shown for each year (1 = 2003, 17 = 2019). Panels **a**–**d** show the regression trend of the effect of sown richness for each of the 17 years (fitting the dependent variable against log species richness. The RYT was also log-transformed prior to analysis. Linear regression relationships are shown on the original scale and the significance for a difference from 0 was two-sided). Inset is the slope of those relationships for each year with the fit statistic (*R*^2^) for the effect of species richness on the biodiversity effects with increasing time, where solid lines highlight significant temporal changes. Since the net, complementarity, and selection effects are measured on a scale of the ANPP (g/m^2^), which declines across the years, in (**e**), we also show the slopes of the effects of species richness on biodiversity effects divided by the average ANPP of all plots for each year.

The relative yield total (RYT), which is the sum of species productivities in mixtures relative to their monocultures, increased with richness (*F*_1, 57.8_ = 50.49, *P* < 0.001), and this positive effect of richness on the RYT significantly increased over the 17 years (log-richness by linear-year interaction: *F*_1, 495.2_ = 7.33, *P* = 0.007, Fig. 2a and Table S6). Species richness also increased the net biodiversity effect (NE, being the difference in a mixture’s ANPP and the average monoculture ANPP: *F*_1, 57.3_ = 40.9, *P* < 0.001). The NE varied among years (*F*_15, 872.5_ = 10.17, *P* < 0.001), but did not show a significant species richness by linear-year interaction (*F*_1, 290.1_ = 10.17, *P* = 0.318, Table S6). However, richness–NE slopes showed a declining trend over the years when the slopes were regressed against the experimental year due to the declining overall productivity over the years (Fig. 2b). Greater species richness increased the CE (*F*_1, 57.4_ = 39.87, *P* < 0.001, Fig. 2c and Table S6) and decreased the SE (*F*_1, 61.3_ = 22.04, *P* < 0.001, Fig. 2d and Table S6). The CE and SE did not vary significantly among years (factor-year effect: *F*_14, 746.6_ = 1.42, *P* = 0.140 and *F*_14, 677.4_ = 0.92, *P* = 0.540, respectively, Table S6), and their relationships with richness did not significantly increase or decrease over the years (log-richness by linear-year interaction: *F*_1, 417.7_ = 0.01, *P* = 0.932 and *F*_1, 397.7_ < 0.01, *P* = 0.975, respectively, Fig. 2c, d and Table S6). Because biodiversity effects are measured on the scale of ANPP (g/m^2^), which declined across the years, accounting for the overall ANPP decline in the field over time by dividing the richness–biodiversity effects slopes by the average ANPP of all plots in each year revealed that on this relative scale the richness–NE and richness–CE relationships did significantly increase and the richness–SE relationships did significantly decrease over the 17-year period (Fig. 2e). To link CE and SE with species asynchrony, population stability, and community stability we calculated these indices over sequential 5-year rolling windows (see Methods, results using 3-year rolling windows were very similar and are presented in the Supplementary Information). This also allowed us to see if the annual SEs scaled up to a 5-year interannual CE^29^. The 5-year CE (see Methods) was significantly correlated with the average annual CE over the same 5 years (Spearman’s rho = 0.655, *P* < 0.001) and the 5-year SE was significantly correlated with the average annual SE (Spearman’s rho = 0.380, *P* < 0.001), indicating that the 5-year CE and SE are reflective of the annual CE and CE. Contrary to expectation, however, the 5-year CE and the average annual SE over the same 5 years were negatively correlated (Spearman’s rho = −0.261, *P* < 0.001).

### Temporal change in diversity–stability relationships and their components

**Fig. 3:**
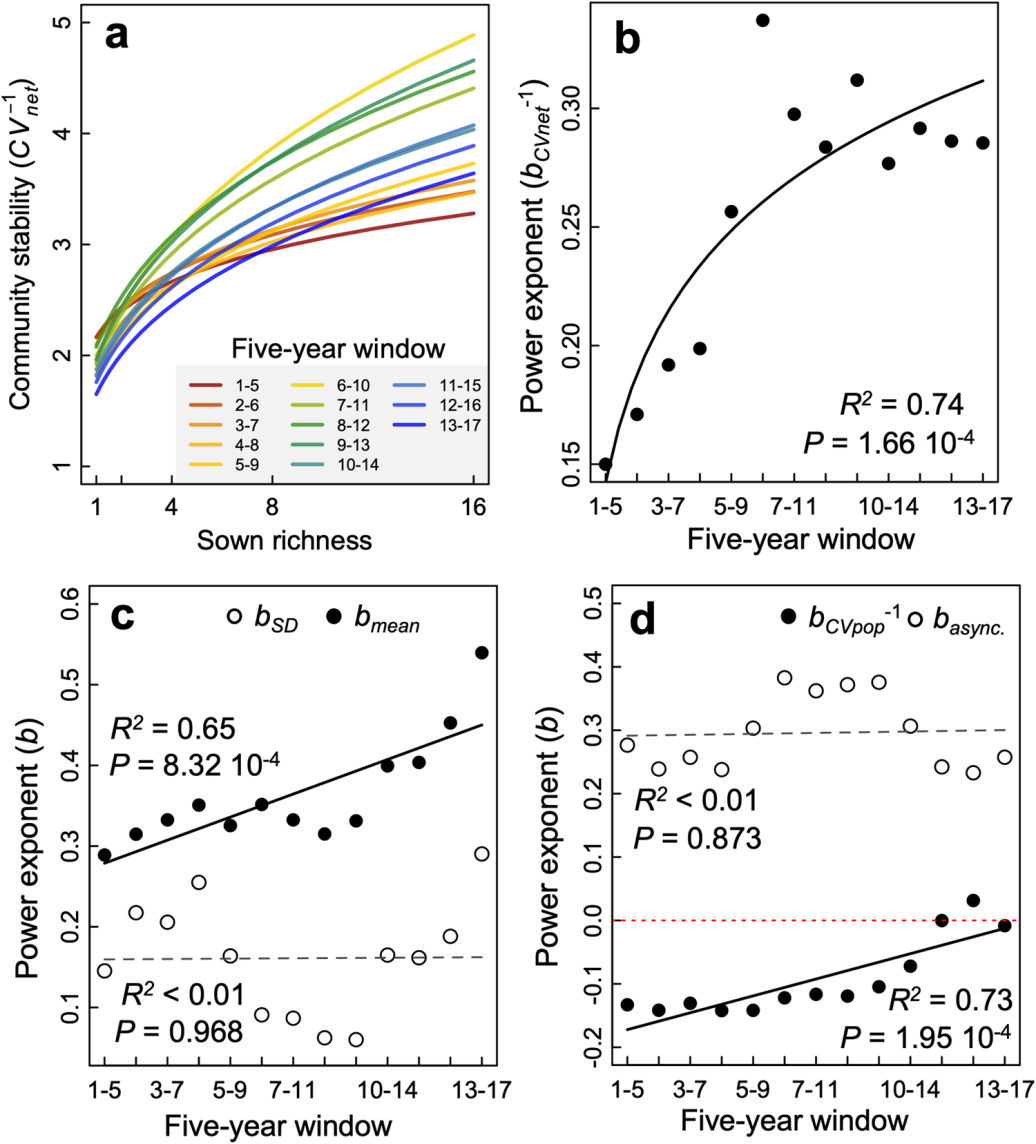
Effects of species richness on community stability and its underlying components. In **a** the richness-community stability (CV_net_^−1^), relationships are sown for each 5-year window indicated by different colors (1 = 2003, 17 = 2019). **b** The change in the slope of the log–log relationship between richness and community stability (power exponent *b* of curves shown in **a** for each consecutive 5-year rolling window. The solid regression line was fit using the relationship slope~log(window). Similarly, **c** are the regression coefficients of richness on the five-year temporal mean and SD in community productivity and **d** on the population stability (CV_pop_^−1^) and asynchrony (async.) of the log–log relationships. These coefficients are relative effects of richness on community stability as *b*_mean_  − *b*_SD_ and *b*_async_ + *b*_*CV*pop_^−1^ are the slope of the log–log relationship between richness and community stability (*b*_*CV*net_^−1^) shown in **b** (see Methods). Black and dashed regression lines respectively highlight significant and non-significant trends along the rolling windows. Tests for significance are two-sided for a difference from 0.

Pooled over 17 years, species richness increased community stability and species asynchrony but decreased population stability (Fig. S2). The stabilizing effect of richness increased across the 13 five-year rolling windows (log-richness by linear-rolling window interaction *F*_1, 888.0_ = 14.23, *P* < 0.001, Fig. 3a and Table S7), but this increasing effect seemed to taper off after the first decade (Fig. 3b). By partitioning the relative effects of species richness on reducing the temporal standard deviation (${b}_{{{{{{{\mathrm{SD}}}}}}}}$) and increasing the temporal mean productivity (${b}_{{{{{{{\mathrm{mean}}}}}}}}$) we found that the latter significantly increased over time (Fig. 3c). Conversely, the richness–${b}_{{{{{{{\mathrm{SD}}}}}}}}$ relationship oscillated through time and did not show any significant directional trend (Fig. 3c). Thus, greater species richness had an increasing effect on stabilizing the community ANPP because of the increasingly positive effect of richness on maintaining a greater 5-year mean ANPP through time compared with their respective monocultures.

Population stability (CV_pop_^−1^) had a negative relationship with species richness (*F*_1, 74.0_ = 4.97, *P* = 0.029), but this effect became less negative across the 5-year rolling windows toward richness having little effect on population stability (log-richness by linear-rolling window interaction *F*_1, 888.0_ = 17.43, *P* < 0.001, Fig. 3d and Table S7). The slope of the richness–asynchrony relationship was positive and did not decline over the five-year rolling windows (*F*_11, 888.0_ = 2.11, *P* = 0.017, Fig. 3d and Table S7). Thus, the temporally increasing effect of species richness on community stability can be attributed to the waning negative richness–population stability relationship while the richness–asynchrony relationship continued to exert its positive effect (Fig. 3d).

### Linking community stability to asynchrony, population stability, and biodiversity effects

**Fig. 4:**
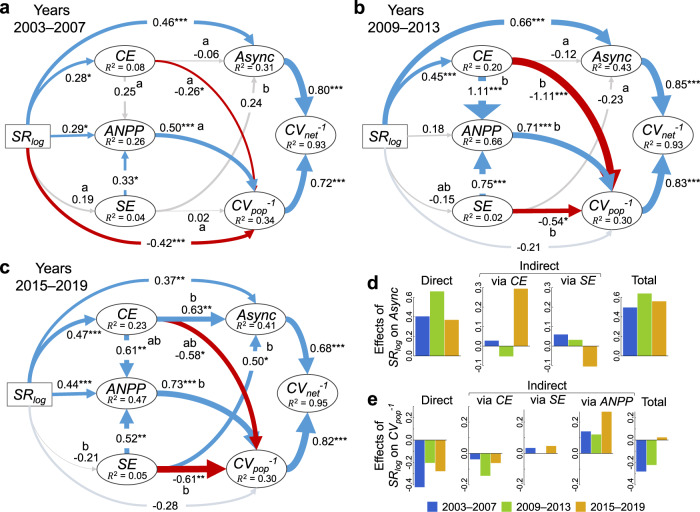
Linking temporal changes in biodiversity effects with stability over three non-overlapping 5-year windows. The structural equation model shows the species richness (SR_log_) effects on the 5-year community productivity (ANPP) and indirectly through the 5-year complementarity (CE) and selection (SE) effects that together affect species population stability (CV_pop_^−1^) and asynchrony (Async). Standardized path coefficients are indicated by arrows with significant positive effects in blue and negative in red. Significance is indicated by *P < 0.05, **P < 0.01, and ***P < 0.001. Different letters adjacent to coefficients indicate significant differences between models **a**–**c** (*P* < 0.05, no multiple comparison adjustments made). Async and CV_pop_^−1^ were allowed to covary, as well as the CE and SE. Fit statistics for the multigroup structural equation model: *Χ*^2^ = 16.1, *P* = 0.375; RMSEA = 0.035, *P*_RMSEA_ *=* 0.524. In **d** and **e**, the direct effects, indirect effects, and the total summed effect, of species richness on asynchrony and population stability are shown, respectively. Tests for significance are two-sided for a difference from 0.

Results of the multigroup structural equation model revealed that the underpinning mechanisms behind the impact of species richness on community stability varied depending on the 5-year window (Fig. 4 and Table S10). Specifically, the CE had a strong significant positive effect on asynchrony in the last 5-year window (Fig. 4c), which differed significantly from the first decade of the experiment, where CE had no significant relationship with asynchrony (Fig. 4a, b). The relationship between the SE and asynchrony also differed among the three independent 5-year windows, where the SE only had a significant positive effect on asynchrony during the first and last 5-year windows (Fig. 4a, c), which differed significantly from the 2009–2013 5-year window (Fig. 4b). Consequently, richness had the strongest positive effect on asynchrony through increasing the CE during the last 5-year window (Fig. 4c), and a lesser indirect negative effect on asynchrony through the SE (Fig. 4d). During this 2015–2019 5-year window the direct effect of richness and the indirect effect of richness through the CE on asynchrony were similarly positive and together drove the positive effect of richness on asynchrony (Fig. 4d). Thus, only after the first decade of the experiment did the effect of richness through the CE start to play a prominent role in driving species asynchrony (also see Fig. S5 for 3-year windows).

The CE had a significant negative effect on the population stability in all three non-overlapping 5-year windows, with the strongest effect occurring during 2009–2013 (Fig. 4a, b). The SE had a significant negative relationship with the population stability during the 2009–2013 and 2015–2019 windows (Fig. 4b, c), which differed from the first 5 years where the SE had no effect on population stability (Fig. 4a). The effect of species richness on the population stability during the first 5-year window (2003–2007) was largely driven by its direct effect (Fig. 4e). During the 2009–2013 window both the negative effects of richness directly, and indirectly through the CE, drove the negative effect of richness on the population stability (Fig. 4e). While richness had a direct negative effect on the population stability during the final 2015–2019 window, this was countered by the positive effect of richness on increasing the ANPP.

**Fig. 5:**
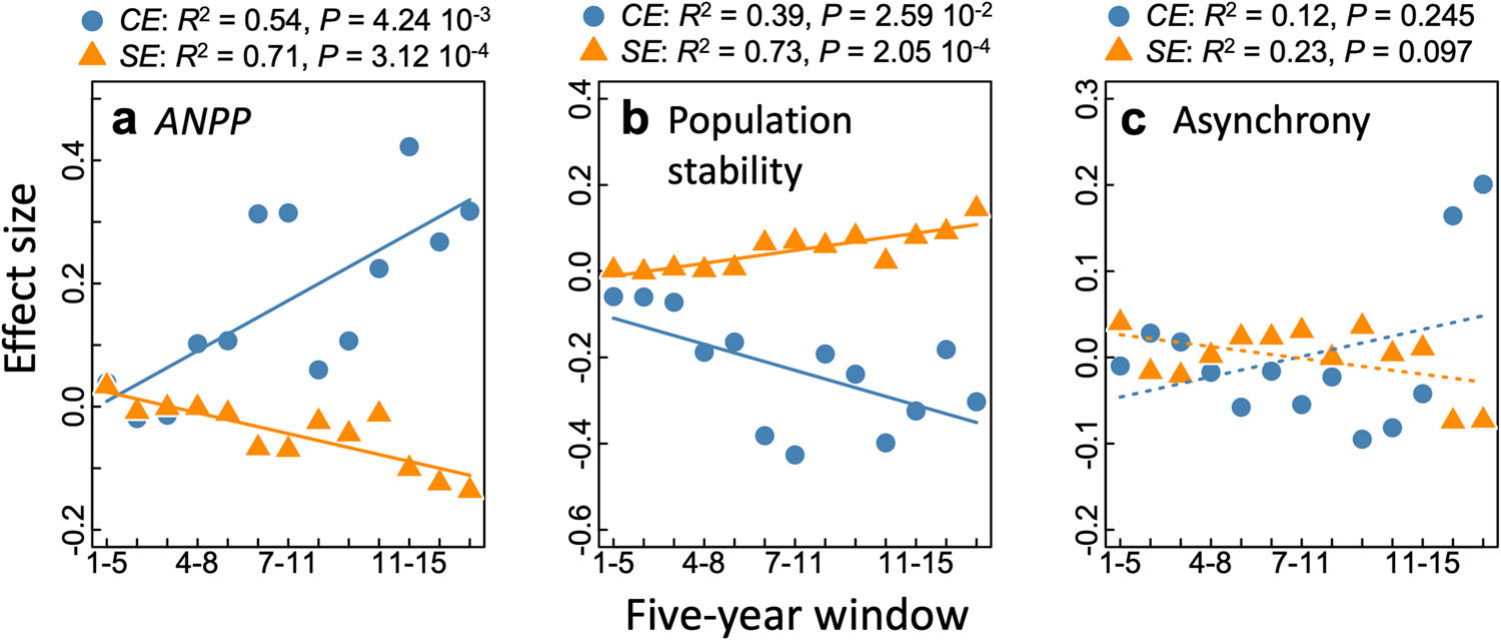
Indirect effects of species richness on community stability through the 5-year complementarity (CE) and selection (SE) effects across 5-year rolling windows. **a** indirect effects through the CE and SE on community stability by their effects on ANPP (richness - > CE/SE - > ANPP - > population stability - > community stability), **b** by their effects on population stability (richness -> CE/SE - > population stability -> community stability), and **c** by their effects on asynchrony (richness -> CE/SE - > Asynchrony -> community stability). Solid lines indicate significant regression trends and dotted lines non-significant trends. Tests for significance are two-sided for a difference from 0. See Fig. S6 for 3-year windows.

Overall, the effect of species richness on community stability increased through time because of the increasing CE in more diverse communities that maintained a greater ANPP (Fig. 5a). However, the effect of richness on community stability through the effect of CE on population stability declined through time (Fig. 5b) and no significant temporal trend in the effect of richness on asynchrony through the CE could be detected (Fig. 5c). The effects of richness on community stability through the SE were also significantly negative through its effect on the ANPP and positive through its effect on the population stability, but changes were not as strong in comparison with the changes in the effects of richness through the CE (Fig. 5a, b).

## Discussion

A growing number of studies have observed that the positive effect of species richness on community productivity (ANPP) can strengthen over time, which can be attributed to a temporally increasing overyielding in more species-rich communities^4,8,10–12,14,16^. Here we further show that this strengthening of the richness–productivity relationship through time results in stronger richness–stability (of community productivity) relationships due to two main mechanisms that also exhibit temporal changes over nearly two decades. First, species richness results in greater community stability over time because of the temporally increasing effect of species richness on productivity through the strengthening effect of richness on the complementarity effect (CE) within 5-year windows. Second, the effect of species richness on destabilizing the population stability weakened, whereas, after a decade, species richness had no effect on the 5-year population stability. Thus, the increasingly positive effect of species richness on community stability became mainly driven by the effects of species richness on species asynchrony within the 5-year windows. Finally, these two mechanisms that lead to greater stability in more diverse mixtures over nearly two decades are not mutually exclusive, because toward the final 5-year window (2015–2019), we found that greater species richness not only influenced asynchrony directly but also indirectly through increasing the five-year complementarity effect (CE). These results show that the underlying mechanisms by which species diversity stabilizes ecosystem functioning themselves can change as the communities develop over time.

The temporally strengthening effect of richness on community stability of productivity occurred via the temporally strengthening effects of richness on mean productivity that occurred due to a strengthening richness–CE relationship. There are several potential mechanisms underlying an increase in the richness–CE relationship through time that maintains greater and more stable productivity in species-rich communities. For instance, changes in diversity–productivity relationships through time have often been thought to be a consequence of deteriorating monoculture performance compared with relatively stable or increasing performance of more species-rich plant communities^10,31–33^. Here we demonstrate that while monoculture productivity declined most rapidly, the rate of declining productivity lessened with each successively higher species richness level (see Fig. 1d). Therefore, in the Jena Experiment, it is the increasing relative decline in productivity with decreasing species richness that strengthened the richness–CE relationship through time and not solely the deterioration of monocultures. It has been hypothesized that a temporal decline in productivity over many years in less species-rich communities could be due to negative plant-enemy feedbacks (i.e., the accumulation of plant species-specific pathogens and herbivores that reduce net productivity)^32,34–36^. Conversely, at the other end of the diversity spectrum in more species-rich communities, greater CE may result from character displacement, where a shift in trait values among co-occurring species occurs over time to avoid resource competition and thus leading to greater complementarity^18,37,38^.

An increasing contribution of the CE to the richness–productivity relationship through time in grassland systems may also be related to the fact that in grassland biodiversity experiments, where local management involves the removal of harvested aboveground biomass without fertilizer addition, soil fertility and plant productivity decrease over time^39^. An increase in the CE as a mechanism behind sustained or increasing diversity effects, therefore, may be partly driven by this temporal reduction in soil fertility in less diverse communities^40^. For instance, as resources are removed from the system over time with the continuous harvesting of aboveground biomass, increasing CEs could be due to the assimilation of atmospheric N_2_ by legumes which may facilitate the N uptake and growth of neighboring non-legume species over time^39,41,42^. Moreover, more diverse plant communities seem to support more efficient soil microbial communities^43^ that maintain soil fertility via soil carbon storage^44–47^ and the reduced leaching of nutrients^48^ and thus closed nutrient cycles^13^. While there are several potential mechanisms by which more diverse communities can maintain relatively greater productivity through temporally increasing CE, where species are, on average able to maintain greater productivity through time in mixtures than if grown in monocultures independently of other species, it is likely that all of these above-mentioned mechanisms are simultaneously at play to drive the increasing importance of diversity for maintaining more stable ecosystem productivity over nearly two decades.

A recent meta-analysis across different terrestrial, aquatic, experimental and observational study systems found that diversity consistently increases stability in ecosystem functioning through increasing species asynchrony, whereas effects via population stability can be positive, neutral, or negative^49^. Coinciding with this observation, we found that although the effect of richness on asynchrony oscillated significantly over the 13 five-year rolling windows, richness consistently had a strong positive effect on species asynchrony with no overall increasing or decreasing trend through time. Conversely, however, while species richness reduced the population stability during the first decade, as has been observed in other experimental biodiversity–stability studies in terrestrial ecosystems^27,34,50^, this negative effect of richness on population stability weakened in the second decade toward richness having little to no effect on population stability. Thus, the community stability became increasingly driven by asynchrony and less by population stability in the second decade of the experiment.

Population stability is comprised of the average temporal standard deviation in species productivity weighted by the net productivity of the community^25^. In our case, the initial negative effect of richness on population stability was due to richness resulting in a greater increase in the temporal standard deviation relative to its effect on productivity. However, the richness–population stability relationship weekend toward neutral in time as the richness–productivity relationship became increasingly positive. This means that eventually, the positive effect of richness on ANPP balanced off the negative effect of richness on increasing species temporal variation in productivity. Taken together, this indicates that species richness had a generalizable effect on increasing the asynchrony within any given 5-year window. However, the increasing positive effect of richness on productivity, via increasing complementarity (CE) among species within a five-year window, countered any destabilizing effect of population stability. While in observational diversity–stability studies, the effect of richness on population stability is generally positive, it is generally negative in experimental studies^49^. This suggests that our experimental plant communities are trending toward a richness–population stability relationship of natural systems as the plant species establish and respond to one another and their local environment for over a decade. However, whether this effect of richness on population stability will eventually progress to being significantly positive will require additional years of observation, highlighting the value of the few existing long-term studies.

Importantly, a notable finding of our study is that only after the first decade did the 5-year CE begin relating to asynchrony. This implies that there is a type of temporal insurance effect of diversity that had developed after the first decade, where interannual complementarity drives the interannual asynchrony in species productivity^30^. Therefore, only after the first decade of the experiment did species in more diverse mixtures in our study become increasingly complementary among years in their productivity, resulting in a greater temporal asynchrony over a 5-year period. This points to the importance of the complementary dynamics among species across years that can result in a portfolio effect resulting in greater asynchrony^4,51^. These complementary temporal dynamics among species are a mechanism that may take many years to become apparent. There could be several drivers for this, one being year-to-year environmental climatic variations. For instance, the experimental site experienced some exceptionally dry (2003, 2011, 2015, and 2018) and wet years (2007, 2009, and 2010), as well as a major flooding event in 2013, where more diverse communities showed increased resilience post flooding^37,52,53^. However, it has been shown elsewhere that environmental variations seem to play a small role in driving species asynchrony and community stability^49,54^.

In addition to annual climatic variations in our system, it is likely that rapid evolutionary changes occurred through interspecific competition, and plant–soil interactions, leading to natural selection processes^55^. For example, we have previously shown that these plant communities result in species complementarity because of increased character displacement to avoid competition when compared with the same plant community composition that has had no co-occurrence history^18,56,57^. Furthermore, it has also been shown that after over a decade, these plant communities are more resilient to environmental perturbation, such as a major flooding event^37^. This implies that more diverse plant communities are increasingly more stable over time as they undergo co-selection and adapt to their local environment. Indeed, after 10–15 years, most of the plant species have likely undergone at least one or two-generational turnover events, since the average maximum age of these plants is around 4 years^58^. This also makes sense in light that previous studies have shown that greater phylogenetic and functional differences among species can lead to greater ecosystem stability^26,42,59–61^, thus inherently also indicating there is an evolutionary basis for the temporally developing diversity–ecosystem stability relationship.

In one of the longest-running biodiversity experiments (the Jena Experiment) after nearly two decades, we found that greater species richness increasingly maintained greater productivity and greater temporal stability of productivity through increasing species complementarity, providing evidence that plant diversity can maintain greater and more stable productivity and that these effects increase over time^4,5,27,50,62–64^. Furthermore, we could show that the underlying mechanisms of community stability, namely species asynchrony and population stability, and overyielding-related complementarity, were also temporally dynamic. Over the 17 years of the experiment, asynchrony and complementarity underpinned diversity effects on stability, whereas population stability played an increasingly less important role. As the communities developed over time, the influence of these mechanisms may have changed due to demographic changes in species populations, including natural selection processes, changes in abiotic and biotic environmental conditions, including resource depletion and build-up of enemy populations and larger-scale perturbations such as a flooding event. It could well be that these temporal changes lead to experimental communities that function more like natural communities that have undergone such temporal development over even longer timespans. Considering that biodiversity effects on stabilizing ecosystem functioning can take well over a decade to develop, it will be important to further assess how asynchrony, population stability, and overyielding-related complementarity continue to support ecosystem stability into the future as the climate and species–species and species–environment interactions continue to change.

## Methods

### Experimental design and data collection

The experiment was set up in 2002 in Jena, Germany, at a site located near the Saale River (50°55′ N, 11°35′ E; 130 m above sea level). The experimental design and field site details are described elsewhere^13^; also see www.the-jena-experiment.de). In brief, the site had been previously used as arable land for more than four decades, but in 2001, the year before the experimental setup, the field was tilled every 2 months and treated with glyphosate in July 2001. A total of 60 plant species typical of local grasslands were selected, including 12 legumes, 16 grasses, 20 tall herbs, and 12 small herbs (Table S1). The experiment consists of 74 large main plots (originally 20 × 20 m in size, in 2010 reduced to 6 × 6 m) set up in four blocks at increasing distances to the Saale River. Plots were sown in a diversity gradient of 1, 2, 4, 8, or 16 plant species crossed with a gradient of functional-group richness ranging from 1 to 4, i.e., including plots of single functional groups ranging in species richness from 1 to 16 (1 to 8 for legumes and small herbs; see Table S2). All species-richness levels had 16 different species compositions as biological replicates, except for the 16-species mixture, which had only 14 different species compositions (no mono-functional-group mixtures of legumes or small herbs could be established at this level). While some species were lost from plots over the years, the weeding ensured that a species richness gradient was maintained based on the initially sown richness (Fig. S2). The plots with different species richness were equally spread across the four blocks. All plant species were also sown as monoculture in plots of 3.5 × 3.5 m (1 × 1 m from 2009 onwards). All plant communities were sown at a density of 1000 germinable seeds per m^2^, with species in mixtures being sown in equal proportions. Two large monoculture plots were abandoned after some years (*Bellis perennis* in 2005; *Cynosurus cristatus* in 2008) because the species were barely present on these plots.

The plant communities were maintained by manual weeding twice per year in early spring (April) and mid-summer (July). From 2010 onward, an additional weeding was done in autumn (late September). In late spring (end of May) and late summer (end of August), standing plant biomass was harvested 3 cm above the soil surface within four randomly positioned 0.5 × 0.2 m quadrats in the large plots and two quadrats of the same size in the small monoculture plots. With the reduction of the size of the plots, the number of quadrats from which biomass was sampled was also reduced to half the number in 2009. At all harvests, except for the summer harvest of 2004, harvested plant material was sorted by species, dried at 70 °C for a minimum of 48 h and weighed by species. In 2004 only the pooled biomass of the sown species was collected in August. After plant material had been collected, the plots were mown to approximately 5 cm above the soil surface at each harvest and the mown plant material was removed. Two biomass harvests per year are the typical management regime of extensively used grasslands in the region. For all following analyses, the biomass data were pooled by year (sum of spring and summer biomass) to assess the aboveground net primary productivity (ANPP) of the communities from 2003 to 2019.

### Calculation of biodiversity effects

We additively partitioned annual net biodiversity effects (NEs) into annual complementarity effects (CEs) and selection effects (SEs) following the additive partitioning method^19^. The additive partitioning is based on the relative yields of the individual plant species in a mixture: ${{{{{{{\mathrm{RY}}}}}}}}_{i}=\frac{{O}_{i}}{{M}_{i}}$, where ${O}_{i}$ is the observed productivity of species *i* in the mixture and ${M}_{i}$ is the productivity of the same species in the monoculture. We first calculated overyielding as the relative yield total ${{{{{{\mathrm{RYT}}}}}}}=\,\sum {{{{{{{\mathrm{RY}}}}}}}}_{i}$. This essentially is the complementarity effect (CE), but on a relative scale, since the complementarity effect is the RYT weighted by the average productivity of those species in monoculture: ${{{{{{\mathrm{CE}}}}}}}=({{{{{{\mathrm{RYT}}}}}}}-1)(\sum \frac{{M}_{i}}{N})$, and the selection effect is calculated as ${{{{{\mathrm{SE}}}}}}=(N-1){{{{{\mathrm{cov}}}}}}({M}_{i},\,{{{{{\mathrm{RY}}}}}}_{i}-1/N)$, where *N* is the number of species in a mixture. The sum of CE and SE equals NE, which is the difference in the observed productivity of the mixture from the average of the respective plant species in monoculture: ${{{{{{\mathrm{NE}}}}}}}=\sum {O}_{i}-\,\sum \frac{{M}_{i}}{N}$. However, since ${{{{{{{\mathrm{RY}}}}}}}}_{i}$ is dependent on the performance of the respective species in monoculture, it is not possible to determine the CE and SE when a species is unable to establish as a monoculture (i.e., ${M}_{i}$ cannot be 0 in the calculation of ${{{{{{{\mathrm{RY}}}}}}}}_{i}$). Therefore, the CE and SE were calculated by excluding species that did not establish in monoculture in either the spring or summer harvests of any specific year^17^. Furthermore, extremely small values in the monoculture productivity of a single species can inflate the complementarity effect and the inclusion of the top three most extreme values strongly influenced the ANOVA model outcomes (see Table S7) and skewed the distribution of the residuals. Accordingly, extreme CE and SE outliers (i.e., those caused by extremely large RY_*i*_) were removed if they were more than six times above or below the upper or lower quartile in magnitude^65^. For all mixed-species plots and years for which CE and SE could be calculated, the exclusion of extreme outliers resulted in the removal of around 6% of the CE and SE values.

### Calculation of community stability and species synchrony

We used a 5-year rolling window, resulting in 13 consecutive 5-year windows with three non-overlapping windows, to also assess whether plant species productivity and their temporal asynchrony changed over the 17-year period. For robustness, we also used three-year rolling windows. Results from the five-year and three-year windows were very similar (see Table S9 and Figs. S3–5 for results using three-year windows). For each 5-year window, we calculated the temporal variation in annual net productivity using the coefficient of variation as ${{{{{{{\mathrm{CV}}}}}}}}_{{{{{{{\mathrm{net}}}}}}}}=\,{\mu }_{{{{{{{\mathrm{net}}}}}}}}/{\sigma }_{{{{{{{\mathrm{net}}}}}}}}$, where ${\sigma }_{{{{{{{\mathrm{net}}}}}}}}$ is the standard deviation in productivity over 5 years and ${\mu }_{{{{{{{\mathrm{net}}}}}}}}$ is the 5-year mean. We used the inverse of CV_net_ (CV_net_^−1^), which is frequently used as a measure of “stability”^3^. Species synchrony^66^ was calculated as: $\theta=\sqrt{\frac{{{\sigma }^{2}}_{{{{{{{\mathrm{net}}}}}}}}}{{\left(\mathop{\sum }\limits_{i}^{N}{\sigma }_{i}\right)}^{2}}}$ where *σ*^*2*^_net_ is the temporal variance in ANPP of a community and $\mathop{\sum }\limits_{i}^{N}{\sigma }_{i}$ is the sum of the temporal standard deviations in ANPPs of the species populations within the community. Because this index of synchrony ranges between 0 and 1, we used 1-$\theta$ as the measure of asynchrony. The index of species synchrony is useful as it can be mathematically partitioned out as a component of the variation in community ANPP (${{{{{{{\mathrm{CV}}}}}}}}_{{{{{{{\mathrm{net}}}}}}}}$) because ${{{{{{{\mathrm{CV}}}}}}}}_{{{{{{{\mathrm{net}}}}}}}}=\,\theta \bullet {{{{{{{\mathrm{CV}}}}}}}}_{{{{{{{\mathrm{pop}}}}}}}}$, where ${{{{{{{\mathrm{CV}}}}}}}}_{{{{{{{\mathrm{pop}}}}}}}}$ is the mean temporal variation of population ANPPs of species within the community, calculated by ${{{{{{{\mathrm{CV}}}}}}}}_{{{{{{{\mathrm{pop}}}}}}}}=\frac{\left(\mathop{\sum }\limits_{i}^{N}{\sigma }_{i}\right)}{{\mu }_{{{{{{{\mathrm{net}}}}}}}}}$^25^$.$ The inverse of the mean temporal variation in species ANPP is a measure of population stability (${{{{{{\rm{CV}}}}}}_{{pop}}}^{-1}$).

We determined the effect of species richness on stabilizing ANPP through the relative effects of richness on maintaining a greater temporal mean and reducing the temporal standard deviation in ANPP. This was done by calculating the power coefficients of the functions $\log ({\mu }_{{{{{{\mathrm{net}}}}}}}) \sim {b}_{{{{{{\mathrm{mean}}}}}}}\cdot \,\log ({{{{{\mathrm{richness}}}}}})$ and $\log ({\sigma }_{{{{{{\mathrm{net}}}}}}}) \sim {b}_{{{{{{\mathrm{SD}}}}}}}\cdot \,\log ({{{{{\mathrm{richness}}}}}})$, where ${b}_{{{{{{{\mathrm{mean}}}}}}}}$ is the relative effect of richness on the temporal mean, and ${b}_{{SD}}$ is the relative effect of richness on the temporal standard deviation. These are the relative effects of species richness on the temporal mean and standard deviation that determine ${b}_{{{{{{{{\mathrm{CVnet}}}}}}}}^{-1}}={b}_{{{{{{{\mathrm{mean}}}}}}}}-{b}_{{{{{{{\mathrm{SD}}}}}}}}$, where ${b}_{{{{{{{{\mathrm{CVnet}}}}}}}}^{-1}}$ is derived from the power function $\log ({{{{{{{\mathrm{CV}}}}}}}_{{{{{{\mathrm{net}}}}}}}}^{-1})={b}_{{{{{{\mathrm{CVnet}}}}}}{}^{-1}}\cdot \,\log ({{{{{\mathrm{richness}}}}}})$^67^. Similarly, we partitioned the relative effects of species richness on asynchrony and population stability, where ${b}_{{{{{{{\mathrm{CVnet}}}}}}}}={b}_{{{{{{{\mathrm{async}}}}}}}.}+{b}_{{{{{{{{\mathrm{CVpop}}}}}}}}^{-1}}$ using the functions $\log ({{{{{\mathrm{asynchrony}}}}}}) \sim {b}_{{{{{{\mathrm{async}}}}}}}\cdot \,\log ({{{{{\mathrm{richness}}}}}})$ and $\log ({{{{{\mathrm{CVpop}}}}}}^{-1}) \sim {b}_{{{{{{\mathrm{CVpop}}}}}}^{-1}}\cdot \,\log ({{{{{\mathrm{richness}}}}}})$.

### Data analysis

All data analyses were done with R version 3.2.4 (http://www.R-project.org). All mixed-effects ANOVA models were calculated using the ASReml package for R (VSN International Ltd., Herts, UK) and the R package pascal (available at: https://github.com/pascal-niklaus/pascal). For all mixed-effects models assessing responses across years, the temporal autocorrelation of residuals across sequential years was included, and the block and plot were included as random-effect terms^68^. The ANPP, annual NE, CE, SE, and RYT, were assessed for relationships with species richness (log-transformed), year as linear followed by a year as a factor and the interactions with richness as fixed-effects terms. The ANPP was square root transformed prior to analysis to meet assumptions of homoscedasticity. Since ANPP varies from year to year, making it difficult to compare the absolute effects of richness on productivity across years, we also assessed the effect of species richness on relative yield (RY), which was calculated by dividing the annual productivity of plots by the mean productivity of all monocultures in that year^8^. The richness–RY relationships were assessed as mentioned above for ANPP and biodiversity effects but using the power function log(RY) ~ log(richness), with year (factor) and the year by log-richness interaction as fixed-effect terms following^8^. The slope coefficients from this log–log regression (power exponent *b*) were extracted from the model and regressed against year (as a linear term) to determine whether the effects of richness on the RY showed a trend over the 17-year period. Similarly, we also regressed the slopes of the effects of richness on NE, CE, SE, and RYT against year as a linear term. Because the biodiversity effects CE, SE, and NE are also measured on the absolute scale, we divided the richness–biodiversity effect slope coefficients by the average ANPP of all plots within the field for each year to express the richness–biodiversity effect slopes relative to the yearly ANPP across the field.

To further understand the temporal changes in the species richness–productivity relationship, we also assessed the relative change in productivity from year 1 in each plot by dividing the annual productivity of each plot by its productivity in year 1 (2003). The productivity relative to year 1 was log-transformed and assessed as a function of species richness level (factor) and year (linear) and their interaction as fixed terms. This allowed us to compare temporal changes in productivity among different richness levels to specifically assess whether less diverse communities declined in productivity more rapidly than did more species-rich communities.

Community stability, population stability, and asynchrony calculated for each 5-year rolling window were assessed for relationships with richness (log-transformed), sequential 5-year window as linear term followed by the 5-year window as a factor and the interactions with richness as fixed-effects terms with block and plot included as random terms. The community and population stability were log-transformed prior to analyses. The power exponents from the log(response) ~ b*log(richness): ${b}_{{{{{{{\mathrm{CVnet}}}}}}}}$, ${b}_{{{{{{{\mathrm{mean}}}}}}}}$, ${b}_{{{{{{{\mathrm{SD}}}}}}}}$, ${b}_{{{{{{{\mathrm{async}}}}}}}.}$, ${b}_{{{{{{{\mathrm{CVpop}}}}}}}}$, relationships were also regressed against the sequential 13 five-year windows (linear or log-linear time) to assess how the relationships changed over time.

### Linking temporal changes in biodiversity effects with stability

To link the CE and SE with the temporal indices of asynchrony, population stability, and community stability we calculated the CE and SE, as well as the net ANPP, for each of the 5-year windows calculating the CE and SE as mentioned above, but with the biomass of the species summed over each five-year period. It should be noted that the 5-year calculation of biodiversity effects holds a slightly different biological meaning than the annual calculation of biodiversity effects. On the annual scale, biodiversity effects result from their spatial and seasonal growth abilities within a given year and growing season. But over a 5-year window, biodiversity effects can arise from a temporal portfolio effect where different species asynchronously drive the ANPP in different years such that varying yearly selection effects, for example, may scale up to 5-year interannual complementarity effects^30^. We then built a multigroup structural equation model to assess how species richness increasingly stabilized the ANPP over the 17 years using three non-overlapping 5-year windows: 2003–2007, 2009–2013, and 2015–2019 using the R package lavaan^69^. We chose three non-overlapping windows as groups for comparison to illustrate that the direct and indirect effects of richness on stability can differ depending on the age of the plant community with data that are unique to each group. To further show the temporal changes in the direct and indirect effects across time, we also used the 15 consecutive 3-year windows. For each window, we assessed the effects of species richness on the 5-year complementarity and selection biodiversity effects that contribute to the five-year productivity. In turn, the population stability is then driven by the 5-year productivity and species richness^25^. Species richness also drives species asynchrony, and together both species asynchrony and population stability determine the community stability^25^. We included in the models the direct effects of species richness on productivity and the direct effect of CE and SE on population stability. We also included the links between asynchrony and CE and SE because in more diverse communities, species that differ more in their performance among years can result in their temporal complementarity and thus increase asynchrony through such a portfolio effect^20,21^. Community stability, population stability, and 5-year productivity were all log-transformed and 5-year CE was min-max scaled and log-transformed. Because extreme outliers in the 5-year CE and SE persisted to influence the model fit, we assessed the model fit across a gradient of sequentially omitting extreme values until the model first reached an RMSEA value of 0. This occurred after omitting the top nine extreme values (about 3% of observations). The 5-year CE and SE were allowed to covary as well as the asynchrony and population stability. We then ran the model over all 13 consecutive 5-year windows to calculate the indirect effects of richness on community stability through the SE and CE effects on the ANPP, population stability, and asynchrony. These indirect effects were then regressed against time (consecutive windows) to detect any increasing or decreasing trends in their effects. This was also repeated with the 3-year windows.

### Reporting summary

Further information on research design is available in the Nature Portfolio Reporting Summary linked to this article.

## Supplementary information

Supplementary Information File

Reporting Summary

## Data Availability

Annual species biomass data is available at https://figshare.com/articles/dataset/Plant_biomass_data_2003-2019/21512352 Detailed data can be requested at http://the-jena-experiment.de/index.php/data/.

## Code availability

R code is available upon request.
