# Supplementary material for: Biodiversity–stability relationships strengthen over time in a long-term grassland experiment
Source: Nat Commun. 2022 Dec 14;13:7752. doi: 10.1038/s41467-022-35189-2 (PMC9751076; doi:10.1038/s41467-022-35189-2)
Supplement: Supplementary file 1 — Supplementary Information File [file 41467_2022_35189_MOESM1_ESM.pdf]

Supplementary Information

Biodiversity–stability relationships strengthen over time in a long-term grassland experiment

Cameron Wagg*, Christiane Roscher, Alexandra Weigelt, Anja Vogel, Anne Ebeling, Enrica de Luca, Anna Roeder, Clemens Kleinspehn, Vicky M. Temperton, Sebastian T. Meyer, Michael Scherer-Lorenzen, Nina Buchmann, Markus Fischer, Wolfgang W. Weisser, Nico Eisenhauer, Bernhard Schmid*

Table S1. Plant species and their functional group (FG) identities.

Species name	FG	Species name	FG
Alopecurus pratensis	Grass	*Glechoma hederacea*	Short herb
Anthoxanthum odoratum	Grass	*Leontodon autumnalis*	Short herb
Arrhenatherum elatius	Grass	*Leontodon hispidus*	Short herb
Avenula pubescens	Grass	*Plantago lanceolata*	Short herb
Bromus erectus	Grass	*Plantago media*	Short herb
Bromus hordeaceus	Grass	*Primula veris*	Short herb
Cynosurus cristatus	Grass	*Prunella vulgaris*	Short herb
Dactylis glomerata	Grass	*Ranunculus repens*	Short herb
Festuca pratensis	Grass	*Taraxacum officinale*	Short herb
Festuca rubra	Grass	*Veronica chamaedrys*	Short herb
Holcus lanatus	Grass	*Achillea millefolium*	Tall herb
Luzula campestris	Grass	*Anthriscus sylvestris*	Tall herb
Phleum pratense	Grass	*Campanula patula*	Tall herb
Poa pratensis	Grass	*Cardamine pratensis*	Tall herb
Poa trivialis	Grass	*Carum carvi*	Tall herb
Trisetum flavescens	Grass	*Centaurea jacea*	Tall herb
Lathyrus pratensis	Legume	*Cirsium oleraceum*	Tall herb
Lotus corniculatus	Legume	*Crepis biennis*	Tall herb
Medicago lupulina	Legume	*Daucus carota*	Tall herb
Medicago varia	Legume	*Galium mollugo*	Tall herb
Onobrychis viciifolia	Legume	*Geranium pratense*	Tall herb
Trifolium campestre	Legume	*Heracleum sphondylium*	Tall herb
Trifolium dubium	Legume	*Knautia arvensis*	Tall herb
Trifolium fragiferum	Legume	*Leucanthemum vulgare*	Tall herb
Trifolium hybridum	Legume	*Pastinaca sativa*	Tall herb
Trifolium pratense	Legume	*Pimpinella major*	Tall herb
Trifolium repens	Legume	*Ranunculus acris*	Tall herb
Vicia cracca	Legume	*Rumex acetosa*	Tall herb
Ajuga reptans	Short herb	*Sanguisorba officinalis*	Tall herb
Bellis perennis	Short herb	*Tragopogon pratensis*	Tall herb

Table S2. Experimental design where numbers indicate the number of plots per plant functional group richness (FGR) and sown plant species richness (SR) levels.

	FGR				
SR	1	2	3	4	Total
1	16				16
2	8	8			16
4	4	4	4	4	16
8	4	4	4	4	16
16	2	4	4	4	14
Total	34	20	12	12	78

Table S3. Mixed-model ANOVA results for assessing the changes in the species richness–community productivity (square-root transformed) and the species richness–relative yield (log-transformed) relationship (see also Fig. 1a and b). Species richness was always log-transformed (SR_{log}) and the change in the effect of richness was assessed using year as a linear term (Y_{linear}) followed by year as a factor with 17 levels (Y_{factor}). Tests for significance are two-sided.

		Productivity			Relative yield		
Fixed	*DF*	DF_{den}	*F*	*P*	DF_{den}	*F*	*P*
SR_{log}	1	71.3	69.45	$4.08\ 10^{-12}$	71.2	80.62	$2.54\ 10^{-13}$
Y_{linear}	1	440.0	354.30	$< 2\ 10^{-16}$	437.1	65.96	0.076
Y_{factor}	15	1074.8	18.08	$< 2\ 10^{-16}$	1072.1	6.55	$1.42\ 10^{-13}$
$SR_{log} \times Y_{linear}$	1	329.9	8.34	0.004	331.1	44.29	$1.18\ 10^{-10}$
$SR_{log} \times Y_{factor}$	15	1074.8	1.40	0.138	1072.1	2.11	0.008
Random		Var.	SE		Var (10^{-3})	SE (10^{-3})	
Block		0.39	0.84		2.23	4.32	
Plot		8.97	2.07		43.22	9.79	
Residual		20.17	1.18		93.76	5.37	
Year ρ_{AR1}		0.51	0.03		0.490	0.028	

Table S4. Slopes and standard errors (SE) for the slope along with the significance (*P*) for a difference from 0 (no relationship) are provided along with the intercept (Inter.) for the relationship between log-transformed sown species richness and a) the square-root transformed community productivity and b) the log-transformed relative yield. The relationships are shown on the original scale in Fig. 1a. Tests for significance are two-sided for a difference from 0.

	a) Community productivity				b) Relative yield			
Year	Inter.	Slope	*SE*	*P*	Inter.	Slope	*SE*	*P*
2003	21.76	2.48	0.65	$1.34\ 10^{-4}$	0.642	0.105	0.045	0.018
2004	21.91	1.79	0.65	0.006	0.603	0.072	0.044	0.104
2005	15.26	2.39	0.65	$2.38\ 10^{-4}$	0.577	0.131	0.045	0.003
2006	15.33	3.56	0.65	$5.23\ 10^{-8}$	0.614	0.200	0.045	$8.33\ 10^{-6}$
2007	16.91	3.86	0.65	$3.07\ 10^{-9}$	0.652	0.208	0.045	$3.37\ 10^{-6}$
2008	11.26	3.79	0.64	$3.45\ 10^{-9}$	0.628	0.288	0.045	$1.09\ 10^{-10}$
2009	12.70	3.85	0.65	$2.52\ 10^{-9}$	0.550	0.244	0.045	$4.55\ 10^{-8}$
2010	11.16	3.21	0.64	$5.84\ 10^{-7}$	0.673	0.258	0.045	$8.14\ 10^{-9}$
2011	13.18	2.58	0.64	$5.86\ 10^{-5}$	0.702	0.183	0.045	$4.18\ 10^{-5}$
2012	11.60	3.77	0.65	$6.14\ 10^{-9}$	0.534	0.250	0.045	$2.62\ 10^{-8}$
2013	11.62	3.17	0.64	$8.77\ 10^{-7}$	0.717	0.256	0.045	$1.06\ 10^{-8}$
2014	11.29	3.91	0.64	$1.24\ 10^{-9}$	0.675	0.290	0.045	$9.08\ 10^{-11}$
2015	9.80	3.66	0.64	$1.24\ 10^{-8}$	0.673	0.322	0.045	$5.88\ 10^{-13}$
2016	7.58	3.74	0.64	$5.79\ 10^{-9}$	0.566	0.359	0.045	$1.31\ 10^{-15}$
2017	9.05	4.33	0.64	$1.62\ 10^{-11}$	0.592	0.361	0.045	$5.82\ 10^{-16}$
2018	7.77	4.06	0.66	$7.17\ 10^{-10}$	0.531	0.373	0.046	$3.98\ 10^{-16}$
2019	6.41	4.88	0.65	$5.36\ 10^{-14}$	0.488	0.459	0.045	$5.89\ 10^{-25}$

Table S5. Mixed-model ANOVA results for the change in community productivity relative to community productivity in year 1 (2003) (see also Fig. 1d). Richness is a factor (SR_{factor}) and year is linear (Y_{linear}). The model was fit with the intercept fixed at 0 (log(1)) hence the degrees of freedom for richness is 5. Tests for significance are two-sided.

Fixed	*DF*	DF_{den}	*F*	*P*
SR_{factor}	5	30.7	17.64	$1.93\ 10^{-4}$
Y_{linear}	1	244.3	211.9	$< 2\ 10^{-16}$
$SR_{factor} \times Y_{linear}$	4	244.3	13.79	$3.73\ 10^{-10}$
Random		Var.	SE	
Block		0.041	0.046	
Plot		0.191	0.051	
Residual		0.629	0.036	
Year ρ_{AR1}		0.498	0.028	

Table S6. Mixed-model ANOVA results for assessing the changes in the species richness–yearly biodiversity effects and the species richness–relative yield total (log-transformed) relationships (see also Fig. 2a–d). Species richness was always log-transformed (SR_{log}) and the change in the effect of richness was assessed using year as a linear term (Y_{linear}) followed by year as a factor with 17 levels (Y_{factor}). Outliers that were more than 6 times the inner quartile range above or below the upper and lower quartiles were removed prior to analyses. See Table S7 for analyses with outliers included that influenced the ANOVA results of the complementarity and selection effects. Tests for significance are two-sided.

	Relative Yield Total (RYT)				*Net effect*			
Fixed	*DF*	DF_{den}	*F*	*P*	*DF*	DF_{den}	*F*	*P*
SR_{log}	1	57.8	50.49	1.81 10^{-9}	1	57.3	40.91	2.84 10^{-8}
Y_{linear}	1	588.9	8.32	0.958	1	474.1	28.91	0.493
Y_{factor}	14	732.5	2.57	0.001	15	872.5	10.17	< 2 10^{-16}
$SR_{log} \times Y_{linear}$	1	495.2	7.33	0.007	1	290.1	1.00	0.318
$SR_{log} \times Y_{factor}$	14	738.2	0.55	0.901	15	872.8	1.41	0.135
Random		Var. 10^{3}	SE 10^{3}			Var. 10^{-3}	SE 10^{-3}	
Block		9.46	15.35			0.59	0.99	
Plot		97.84	26.93			4.97	1.88	
Residual		529.67	27.14			33.55	1.99	
Year ρ_{AR1}		0.120	0.039			0.44	0.03	

	Complementarity effect				*Selection effect*			
Fixed	*DF*	DF_{den}	*F*	*P*	*DF*	DF_{den}	*F*	*P*
SR_{log}	1	57.4	39.87	3.87 10^{-8}	1	61.3	22.04	1.27 10^{-5}
Y_{linear}	1	623.1	4.02	0.449	1	606.1	1.05	0.673
Y_{factor}	14	746.6	1.42	0.140	14	677.4	0.92	0.540
$SR_{log} \times Y_{linear}$	1	417.7	0.01	0.932	1	397.7	0.00	0.975
$SR_{log} \times Y_{factor}$	14	750.5	0.91	0.545	14	685.8	0.95	0.500
Random		Var. 10^{-4}	SE 10^{-4}			Var. 10^{-4}	SE 10^{-4}	
Block		-0.48	0.53			<0.01	<0.01	
Plot		10.33	3.17			9.52	2.69	
Residual		79.04	3.98			59.81	3.14	
Year ρ_{AR1}		0.09	0.04			0.06	0.04	

Table S7. Mixed-model ANOVA results with (a) no outliers removed and (b) the top three most extreme outliers removed for assessing the changes in the species richness–yearly biodiversity effects and the species richness–relative yield total (log-transformed) relationships. Species richness was always log-transformed (SR_{log}) and the change in the effect of richness was assessed using year as a linear term (Y_{linear}) followed by year as a factor with 17 levels (Y_{factor}). Tests for significance are two-sided.

a)	*Complementarity effect*				*Selection effect*			
Fixed	*DF*	DF_{den}	*F*	*P*	*DF*	DF_{den}	*F*	*P*
SR_{log}	1	57.7	0.07	0.795	1	343.1	0.01	0.926
Y_{linear}	1	674.2	0.01	0.717	1	766.6	0.00	0.772
Y_{factor}	14	797.5	1.25	0.232	14	783.0	1.42	0.136
$SR_{log} \times Y_{linear}$	1	440.6	0.03	0.873	1	514.6	0.03	0.853
$SR_{log} \times Y_{factor}$	14	798.3	0.17	1.000	14	788.0	0.22	0.999
Random		Var. 10^8	SE 10^8			Var. 10^8	SE 10^8	
Block		-0.08	0.28			0.00	0.00	
Plot		-0.11	1.31			0.00	0.00	
Residual		102.86	4.88			110.68	5.27	
Year ρ_{AR1}		-0.001	0.035			-0.002	0.034	

b)	*Complementarity effect*				*Selection effect*			
Fixed	*DF*	DF_{den}	*F*	*P*	*DF*	DF_{den}	*F*	*P*
SR_{log}	1	57.8	6.56	0.013	1	64.7	5.25	0.028
Y_{linear}	1	664.4	3.61	0.147	1	670.2	2.99	0.139
Y_{factor}	14	789.0	1.47	0.117	14	735.1	1.49	0.109
$SR_{log} \times Y_{linear}$	1	439.8	0.02	0.883	1	430.1	0.08	0.774
$SR_{log} \times Y_{factor}$	14	789.3	0.54	0.910	14	742.6	0.43	0.967
Random		Var. 10^8	SE 10^8			Var. 10^{-4}	SE 10^{-4}	
Block		-0.03	0.02			0.00	0.00	
Plot		0.19	0.14			19.74	15.53	
Residual		8.93	0.42			964.40	47.64	
Year ρ_{AR1}		-0.036	0.036			-0.037	0.038	

Table S8. Mixed-model ANOVA results of the analysis of the community stability, population stability and asynchrony. The log of species richness is a linear term (SR_{log}) along a five-year rolling window (13 consecutive 5-year windows) as linear (W_{linear}) to assess components of stability changed through time followed by window as a factor (W_{factor}) to assess whether effects varied among the five-year windows (see also Fig. 3). Community and population stability was log-transformed prior to analyses. Tests for significance are two-sided.

		Community stability (CV_{net}^{-1})		
Fixed	*DF*	DF_{den}	*F*	*P*
SR_{log}	1	74	44.99	1.23 10^{-9}
W_{linear}	1	888.0	0.13	0.008
W_{factor}	11	888.0	2.66	0.002
$SR_{log} \times W_{linear}$	1	888.0	14.23	1.16 10^{-5}
$SR_{log} \times W_{factor}$	11	888.0	0.46	0.929
Random		Var 10^{3}	SE 10^{3}	
Block		<0.01	<0.01	
Plot		89.94	16.68	
Residual		149.20	7.08	
		Population stability (CV_{pop}^{-1})		
Fixed	*DF*	DF_{den}	*F*	*P*
SR_{log}	1	74.0	4.97	0.029
W_{linear}	1	888.0	0.83	0.019
W_{factor}	11	888.0	0.89	0.553
$SR_{log} \times W_{linear}$	1	888.0	17.43	3.28 10^{-5}
$SR_{log} \times W_{factor}$	11	888.0	0.48	0.917
Random		Var 10^{-3}	SE 10^{-3}	
Block		0.00	0.00	
Plot		70.02	12.79	
Residual		100.86	4.79	
		Asynchrony ($1\text{-}\theta$)		
Fixed	*DF*	DF_{den}	*F*	*P*
SR_{log}	1	71.2	260.70	< 2 10^{-16}
W_{linear}	1	888.0	0.86	0.272
W_{factor}	11	888.0	2.11	0.017
$SR_{log} \times W_{linear}$	1	888.0	0.29	0.591
$SR_{log} \times W_{factor}$	11	888.0	1.49	0.129
Random		Var 10^{-3}	SE 10^{-3}	
Block		0.49	0.87	
Plot		9.61	1.84	
Residual		17.70	0.84	

Table S9. Mixed-model ANOVA results of the analysis of the community stability, population stability and asynchrony. The log of species richness is a linear term (SR_{log}) along a three-year rolling window (15 consecutive three-year windows) as linear (W_{linear}) to assess components of stability changed through time followed by window as a factor (W_{factor}) to assess whether effects varied among the three-year windows (see also Fig. 3). Population stability was log transformed and community stability and asynchrony were square root-transformed prior to analyses to improve homoscedasticity. Tests for significance are two-sided.

		Community stability (CV_{net}^{-1})		
Fixed	*DF*	DF_{den}	*F*	*P*
SR_{log}	1	73.9	54.20	$1.92\ 10^{-10}$
W_{linear}	1	1033.2	2.50	0.023
W_{factor}	13	1033.9	5.30	$3.24\ 10^{-09}$
$SR_{log} \times W_{linear}$	1	1033.1	10.40	0.001
$SR_{log} \times W_{factor}$	13	1033.0	0.60	0.891
Random		Var 10^{3}	SE 10^{3}	
Block		0.00	0.00	
Plot		5.98	1.30	
Residual		28,36	1.25	
		Population stability (CV_{pop}^{-1})		
Fixed	*DF*	DF_{den}	*F*	*P*
SR_{log}	1	74.0	3.35	0.073
W_{linear}	1	1033.3	4.99	0.002
W_{factor}	13	1033.0	4.62	$8.73\ 10^{-8}$
$SR_{log} \times W_{linear}$	1	1033.2	14.57	$1.43\ 10^{-5}$
$SR_{log} \times W_{factor}$	13	1033.1	0.44	0.956
Random		Var 10^{-3}	SE 10^{-3}	
Block		0.00	0.00	
Plot		63.0	13.0	
Residual		240.1	10.6	
		Asynchrony ($1\text{-}\theta$)		
Fixed	*DF*	DF_{den}	*F*	*P*
SR_{log}	1	71.3	265.60	$< 2\ 10^{-16}$
W_{linear}	1	1033.5	0.20	0.603
W_{factor}	13	1033.1	1.82	0.035
$SR_{log} \times W_{linear}$	1	1033.4	0.02	0.886
$SR_{log} \times W_{factor}$	13	1033.3	1.04	0.408
Random		Var 10^{-3}	SE 10^{-3}	
Block		0.57	0.86	
Plot		6.41	1.54	
Residual		40.79	1.79	

Table S10. Results from the multigroup structural equation model showing the pairwise differences (*Diff*) in coefficients between the groups of non-overlapping five-year windows: 1 = years 2003–2007, 2 = years 2009–2013, 3 = years 2015–2019. Tests for significance are two-sided. No adjustment was made for comparisons among groups.

Effects of richness on:

	CE			*SE*			*ANPP*			Population stability			*Asynchrony*		
Group	*Diff*	*SE*	*P*	*Diff*	*SE*	*P*	*Diff*	*SE*	*P*	*Diff*	*SE*	*P*	*Diff*	*SE*	*P*
1 vs 2	-0.16	0.21	0.324	0.34	0.22	0.053	0.11	0.16	0.267	-0.18	0.21	0.391	-0.21	0.17	0.280
1 vs 3	-0.19	0.22	0.253	**0.41**	**0.23**	**0.026**	-0.15	0.18	0.708	-0.11	0.22	0.624	0.08	0.19	0.809
2 vs 3	-0.03	0.21	0.841	0.07	0.23	0.699	-0.26	0.14	0.070	0.07	0.24	0.778	0.29	0.19	0.223

Effects of CE on:

	ANPP			Population stability			Asynchrony		
Group	*Diff*	*SE*	*P*	*Diff*	*SE*	*P*	*Diff*	*SE*	*P*
1 vs 2	**-0.87**	**0.16**	**<0.001**	**0.86**	**0.30**	**0.003**	0.06	0.18	0.795
1 vs 3	-0.36	0.19	0.269	0.32	0.26	0.216	**-0.69**	**0.23**	**0.006**
2 vs 3	0.51	0.18	0.053	-0.54	0.37	0.110	**-0.75**	**0.25**	**0.006**

Effects of SE on:

	ANPP			Population stability			Asynchrony		
Group	*Diff*	*SE*	*P*	*Diff*	*SE*	*P*	*Diff*	*SE*	*P*
1 vs 2	-0.42	0.15	0.138	**0.57**	**0.25**	**0.023**	**0.47**	**0.17**	**0.024**
1 vs 3	-0.19	0.18	0.719	**0.63**	**0.23**	**0.014**	-0.26	0.21	0.216
2 vs 3	0.23	0.16	0.327	0.06	0.31	0.981	**-0.73**	**0.22**	**0.004**

Effects of ANPP on:

	population stability		
Group	*Diff*	*SE*	*P*
1 vs 2	-0.21	0.27	0.035
1 vs 3	**-0.23**	**0.22**	**0.028**
2 vs 3	-0.02	0.32	0.802

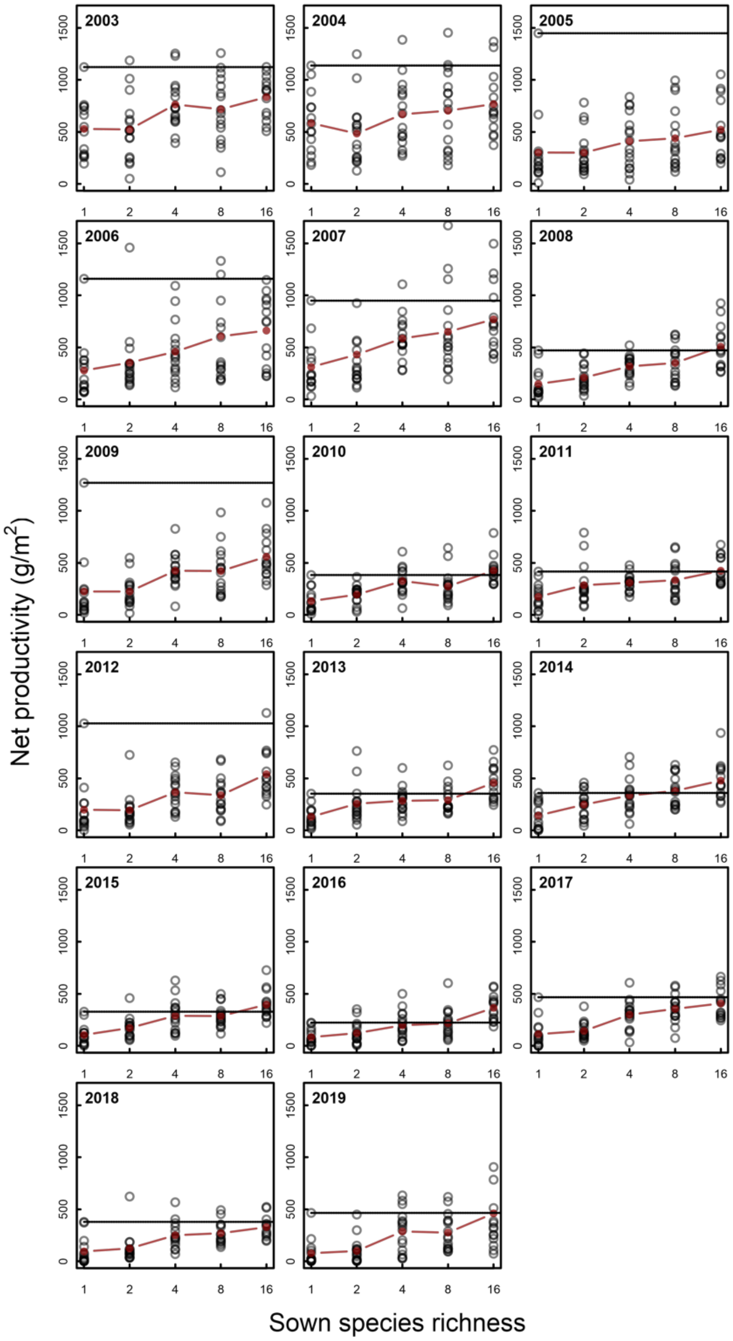

Fig S1. Scatterplots of the raw biomass data for each plot (points) are shown for each of the 17 years where community productivity was collected. The red line highlights the trend between consecutive sown species richness means. The dashed line highlights the most productive monoculture.

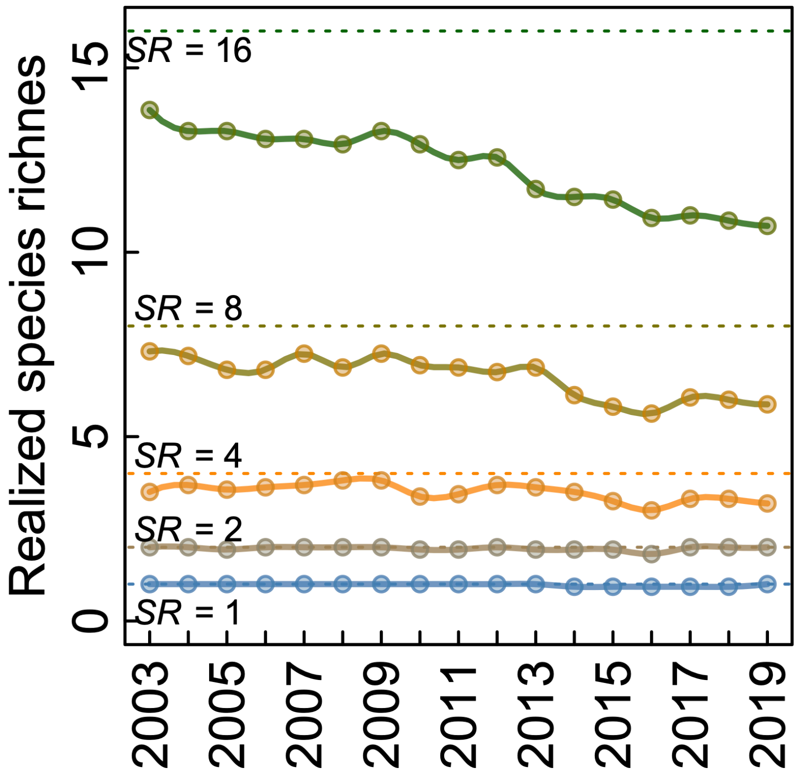

Fig. S2. Change in species realized richness over the 17-year period for each sown richness level. Dotted lines highlight the initial sown species richness (SR).

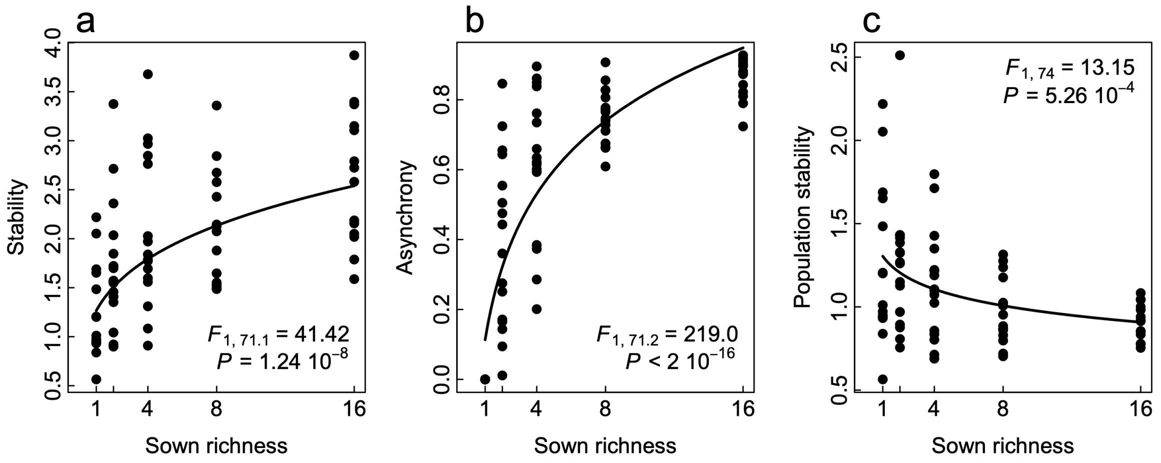

Fig. S3. Scatter plots showing the relationship between sown species richness and (a) stability (CV_{net}^{-1}), (b) asynchrony and (c) population stability (CV_{pop}^{-1}) over the entire 17-year period. Solid lines are the regression trends for the log-log relationship and associated ANOVA results for the relationship are provided. Tests for significance are two-sided.

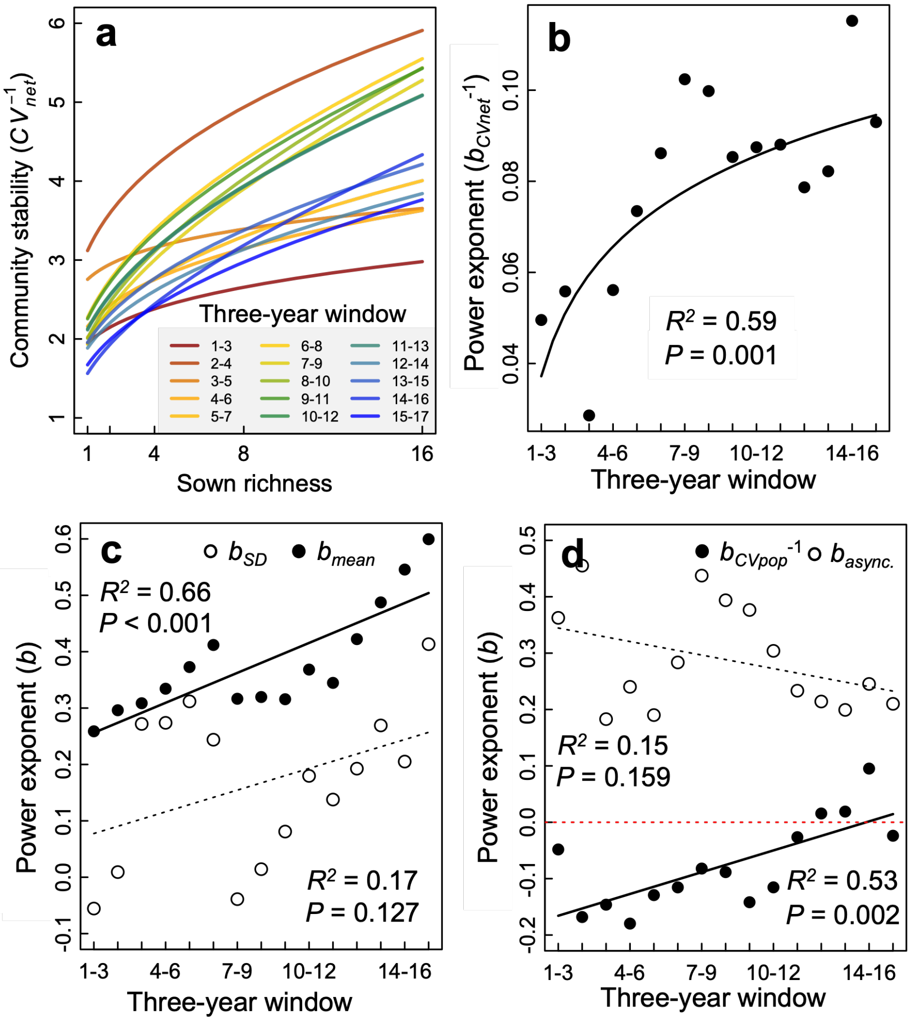

Fig. S4. Effects of species richness on community stability and its underlying components.

In (**a**) the richness-community stability (CV_{net}^{-1}) relationships are sown for each three-year window indicated by different colors (1 = 2003, 17 = 2019). (**b**) The change in the slope of the log–log relationship between richness and community stability (power exponent b of curves shown in **a**) for each consecutive three-year rolling window. The solid regression line was fit using the relationship slope~log(window). Similarly, (**c**) are the regression coefficients of richness on the three-year temporal mean and SD in community productivity and (**d**) on the population stability (CV_{pop}^{-1}) and asynchrony (async.) of the log-log relationships. These coefficients are relative effects of richness on community stability as b_{mean} - b_{SD} and b_{async} + b_{CVpop}^{-1} are the slope of the log–log relationship between richness and community stability (b_{CVnet}^{-1}) shown in (**b**) (see Methods). Black and dashed regression lines respectively highlight significant and non-significant trends along the rolling winnows. Tests for significance are two-sided.

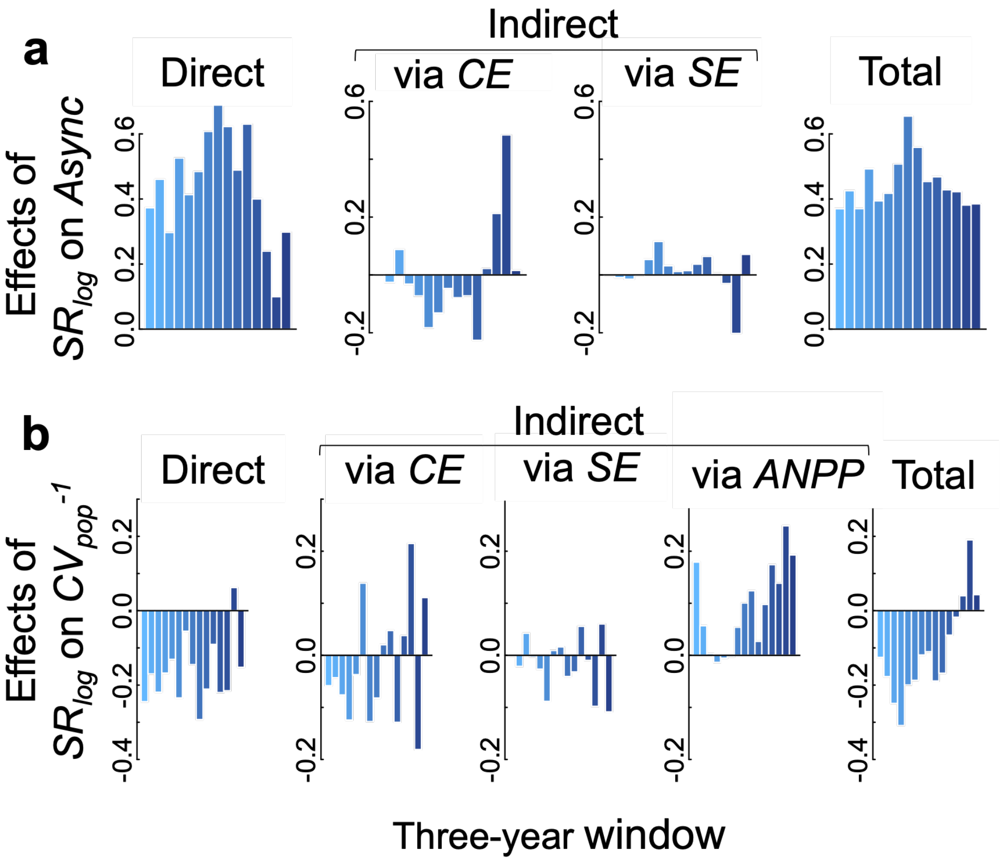

Fig. S5. The direct effects, indirect effects, and the total summed effect, of species richness on **a** asynchrony and **b** population stability for each of the sequential three-year rolling windows (light blue = 2003 – 2005 to dark blue 2017 – 2019).

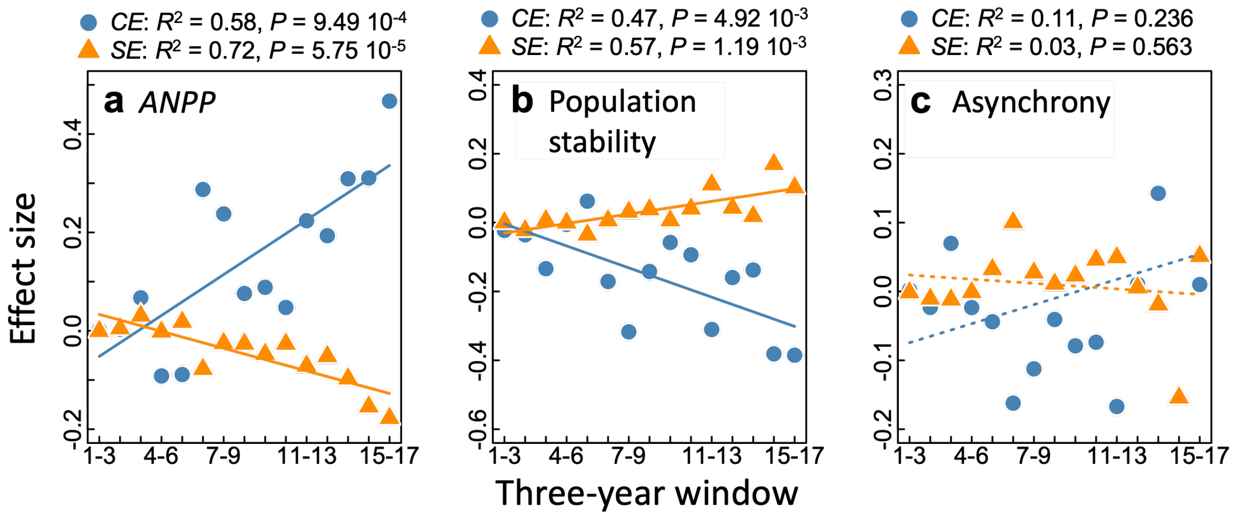

Fig. S6. Indirect effects of species richness on community stability the three-year complementarity (*CE*) and selection (*SE*) effects across three-year rolling windows. **a** indirect effects through the *CE* and *SE* on community stability by their effects on *ANPP* (richness -> *CE*/*SE* -> *ANPP* -> population stability -> community stability), **b** by their effects on population stability (richness -> *CE*/*SE* -> population stability -> community stability), and **c** by their effects on asynchrony (richness -> *CE*/*SE* -> Asynchrony -> community stability). Solid lines indicate significant regression trends and dotted lines non-significant trends. Tests for significance are two-sided for a difference from 0.
