# Supplementary material for: Biodiversity–stability relationships strengthen over time in a long-term grassland experiment
Source: Nat Commun. 2022 Dec 14;13:7752. doi: 10.1038/s41467-022-35189-2 (PMC9751076; doi:10.1038/s41467-022-35189-2)
Supplement: Supplementary file 2 — Reporting Summary [file 41467_2022_35189_MOESM2_ESM.pdf]

nature portfolio

Corresponding author(s): Cameron Wagg

Last updated by author(s): Nov 7, 2022

Reporting Summary

Nature Portfolio wishes to improve the reproducibility of the work that we publish. This form provides structure for consistency and transparency in reporting. For further information on Nature Portfolio policies, see our Editorial Policies and the Editorial Policy Checklist.

Statistics

For all statistical analyses, confirm that the following items are present in the figure legend, table legend, main text, or Methods section.

n/a	Confirmed	
☐	☒	The exact sample size (*n*) for each experimental group/condition, given as a discrete number and unit of measurement
☐	☒	A statement on whether measurements were taken from distinct samples or whether the same sample was measured repeatedly
☐	☒	The statistical test(s) used AND whether they are one- or two-sided<br>*Only common tests should be described solely by name; describe more complex techniques in the Methods section.*
☒	☐	A description of all covariates tested
☐	☒	A description of any assumptions or corrections, such as tests of normality and adjustment for multiple comparisons
☐	☒	A full description of the statistical parameters including central tendency (e.g. means) or other basic estimates (e.g. regression coefficient) AND variation (e.g. standard deviation) or associated estimates of uncertainty (e.g. confidence intervals)
☐	☒	For null hypothesis testing, the test statistic (e.g. *F*, *t*, *r*) with confidence intervals, effect sizes, degrees of freedom and *P* value noted<br>*Give P values as exact values whenever suitable.*
☒	☐	For Bayesian analysis, information on the choice of priors and Markov chain Monte Carlo settings
☒	☐	For hierarchical and complex designs, identification of the appropriate level for tests and full reporting of outcomes
☐	☒	Estimates of effect sizes (e.g. Cohen's *d*, Pearson's *r*), indicating how they were calculated

Our web collection on statistics for biologists contains articles on many of the points above.

Software and code

Policy information about availability of computer code

Data collection	No special software was used to collect data
Data analysis	All data analyses were performed in R for Mac OS X GUI version 4.1.2 (www.R-project.org). The ASReml package for R (v4.1.0154, VSN international) was used mixed effect ANOVA models. Any R code can be made available upon request to the corresponding authors.

For manuscripts utilizing custom algorithms or software that are central to the research but not yet described in published literature, software must be made available to editors and reviewers. We strongly encourage code deposition in a community repository (e.g. GitHub). See the Nature Portfolio guidelines for submitting code & software for further information.

Data

Policy information about availability of data

All manuscripts must include a data availability statement. This statement should provide the following information, where applicable:

- Accession codes, unique identifiers, or web links for publicly available datasets
- A description of any restrictions on data availability
- For clinical datasets or third party data, please ensure that the statement adheres to our policy

Data can be requested at http://the-jena-experiment.de/index.php/data/. Data will be published online eventually at Figshare (https://figshare.com/articles/dataset/Plant_biomass_data_2003-2019/21512352)

Human research participants

Policy information about studies involving human research participants and Sex and Gender in Research.

Reporting on sex and gender	NA
Population characteristics	NA
Recruitment	NA
Ethics oversight	NA

Note that full information on the approval of the study protocol must also be provided in the manuscript.

Field-specific reporting

Please select the one below that is the best fit for your research. If you are not sure, read the appropriate sections before making your selection.

☐ Life sciences ☐ Behavioural & social sciences ☒ Ecological, evolutionary & environmental sciences

For a reference copy of the document with all sections, see nature.com/documents/nr-reporting-summary-flat.pdf

Ecological, evolutionary & environmental sciences study design

All studies must disclose on these points even when the disclosure is negative.

Study description	The study consists of 74 grassland plant communities consisting of 1, 2, 4, 8, or 16 species that were established in 2002. Each species richness level was replicated at least 14 times. Plots are arranged in 4 blocks. The expeerimental design has been published extensively (see Weisser et al. 2017. Basic and Applied Ecology).
Research sample	Aboveground biomass of individual species as has been done in the experiment over nearly two decades (there are 60 species an over 100 publications from this long term project. see http://the-jena-experiment.de/index.php/main-experiment/).
Sampling strategy	Plant aboveground biomass was sampled from four 0.5 x 0.2 m quadrats per plot until 2009 after which only two 0.5 x 0.2 m quadrats were used, sorted to species, dried and weighed. This was done twice per year(Late May and August) from 2003 to 2019. Spring and summer samples were summed as a measure of annual net productivity. Thiswas done for consistency over the 17 years and with the previous 100s of publications using a similar experimental design.
Data collection	The data was collected by various researchers and graduate students over the 17 year period. Data was collected with shearers to cut plants, an oven to dry plants, a balance to weigh dry biomass and a pen and paper to record it. A computer was used with Excell to enter the data.
Timing and spatial scale	Data collection started in 2003 and for this study ended in 2019. Plant biomass sampling was done twice per year (Late May and August) from 2003 to 2019. Data were pooled across seasons within each year for the annual biomass production data.
Data exclusions	No original data were excluded from the data set. Some derived values of indices were excluded due to their calculation resulting in extreme outliers. This is described in detail in the methods.
Reproducibility	The experiment is repeatable as we have repeatedly recorded and analyzed data over 17 years. A similar experiment also is replicated in Cedar Creek, USA.
Randomization	The different species richness level treatments were randomized within each replicate block.
Blinding	Not Applicable because it is a study on plant communities.

Did the study involve field work? ☒ Yes ☐ No

Field work, collection and transport

Field conditions	These have been reported in previous publications on the field experiment. See Weisser et al. 2017, Basic and Applied Ecology and Roscher et al. 2004, Basic and Applied Ecology, 5.
Location	50°55’ N, 11°35’ E; 130 m above sea level
Access & import/export	The experimental site where the plant communities are maintained is owned by the research consortia and the University of Jena.

Disturbance	The plant communities are being maintained continually through weeding as the experiment is still ongoing.

Reporting for specific materials, systems and methods

We require information from authors about some types of materials, experimental systems and methods used in many studies. Here, indicate whether each material, system or method listed is relevant to your study. If you are not sure if a list item applies to your research, read the appropriate section before selecting a response.

Materials & experimental systems

n/a	Involved in the study	
[x]	[ ]	Antibodies
[x]	[ ]	Eukaryotic cell lines
[x]	[ ]	Palaeontology and archaeology
[x]	[ ]	Animals and other organisms
[x]	[ ]	Clinical data
[x]	[ ]	Dual use research of concern

Methods

n/a	Involved in the study	
[x]	[ ]	ChIP-seq
[x]	[ ]	Flow cytometry
[x]	[ ]	MRI-based neuroimaging
